# Review on Tailoring PEDOT:PSS Layer for Improved Device Stability of Perovskite Solar Cells

**DOI:** 10.3390/nano11113119

**Published:** 2021-11-19

**Authors:** Yijie Xia, Guowang Yan, Jian Lin

**Affiliations:** 1 School of Mechanical Engineering, University of Shanghai for Science and Technology, Shanghai 200093, China; ygw_527@163.com; 2 Institute of Photonic Chips, University of Shanghai for Science and Technology, Shanghai 200093, China; jianlin@usst.edu.cn; 3 Centre for Artificial-Intelligence Nanophotonics, School of Optical-Electrical and Computer Engineering, University of Shanghai for Science and Technology, Shanghai 200093, China

**Keywords:** PEDOT:PSS, inverted perovskite solar cells, stability

## Abstract

Poly(3,4-ethylenedioxythiophene):poly(styrene sulfonate) (PEDOT:PSS) has high optical transparency in the visible light range and low-temperature processing condition, making it one of the most widely used polymer hole transport materials inverted perovskite solar cells (PSCs), because of its high optical transparency in the visible light range and low-temperature processing condition. However, the stability of PSCs based on pristine PEDOT:PSS is far from satisfactory, which is ascribed to the acidic and hygroscopic nature of PEDOT:PSS, and property differences between PEDOT:PSS and perovskite materials, such as conductivity, work function and surface morphology. This review summaries recent efficient strategies to improve the stability of PEDOT:PSS in PSCs and discusses the underlying mechanisms. This review is expected to provide helpful insights for further increasing the stability of PSCs based on commercial PEDOT:PSS.

## 1. Introduction

**Figure 1:**
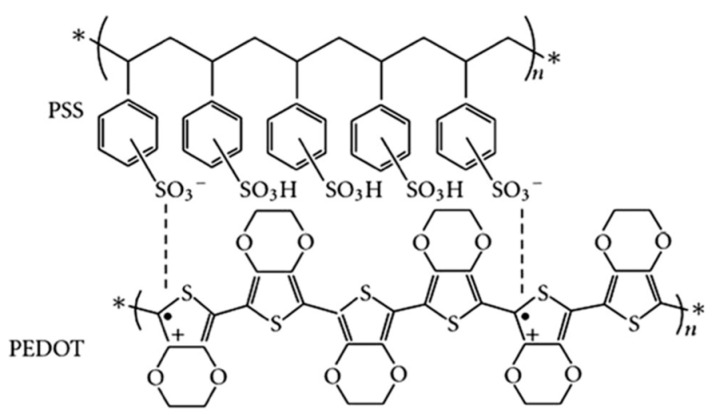
Chemical structure of PEDOT:PSS. n: degree of polymerization, *: repeated structural units, +: positive charge, •: negative charge.

Poly(3,4-ethylenedioxythiophene):poly(styrene sulfonate) (PEDOT:PSS, chemical structure is shown in Figure 1) is the most successful conducting polymer, which has been widely used in displays, transistors, various sensors and photovoltaics (PVs) [1,2,3,4,5,6,7,8,9,10,11,12,13,14,15,16,17]. It can be dispersed in water as well as some organic solvents and conventional solution based coating methods can be used to fabricate high-quality PEDOT:PSS films [18,19]. PEDOT:PSS films are uniform and highly transparent in the visible range. The electrical conductivity of PEDOT:PSS film can be adjusted within 10^−2^ to 10^3^ S/cm with certain synthetic conditions, the utilization of different additives or post-treatment methods [20,21,22,23,24,25,26,27,28,29,30,31,32,33,34,35,36]. Furthermore, PEDOT:PSS is a low cost material with excellent thermal stability and high mechanical flexibility. Therefore, in recent years, PEDOT:PSS is the most popular hole transport layer (HTL) used in inverted perovskite solar cells (PSCs) [37,38,39].

Perovskite solar cells receive much attention as a next-generation solar technology for energy harvesting due to their very impressive energy conversion efficiency with low fabrication cost [40,41,42,43,44,45,46,47,48,49,50,51,52,53,54,55,56,57,58,59,60,61,62,63,64,65,66]. Power conversion efficiencies (PCE) of PSCs are increased from 3.8% in 2009 up to the current world record of 25.6% [67,68]. However, the serious long-term instability of PSCs limits their commercialization. The stability of PSCs, especially under ambient ultraviolet radiation and humidity, is one of the major drawbacks recently addressed by the photovoltaic scientific community. For PSCs devices, HTL is usually indispensable for effectively blocking electrons and transporting holes. In addition, it affects the quality of the upper perovskite layer which directly affects the efficiency and stability of the devices [69,70].

**Figure 2:**
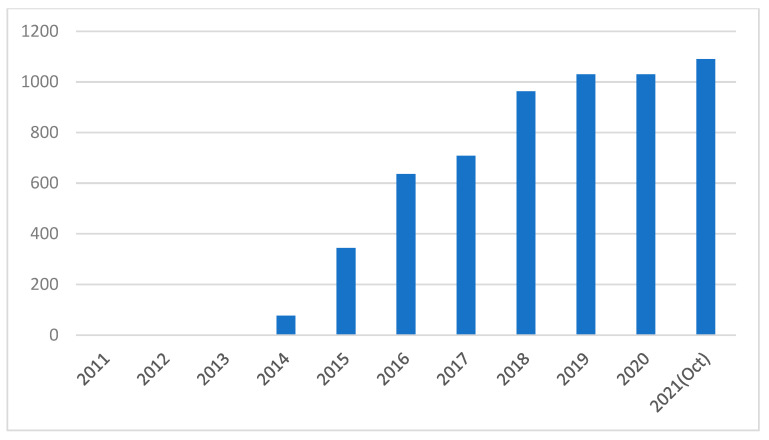
Number of articles published per year on PSCs using PEDOT:PSS [109].

Most inverted PSCs using PEDOT:PSS as the HTL material due to the low temperature processability and simple solution-process ability. Figure 2 shows the increment in the number of research papers published in recent years on PSCs using PEDOT:PSS as the HTL. The increased research indicates that PEDOT:PSS is a promising HTL material in PSCs. However, the use of PEDOT:PSS would affect the stability of cells due to its hygroscopic and acidic nature [71]. The acidic nature of PEDOT:PSS will corrode the ITO electrode. Moreover, the hygroscopic nature of PEDOT:PSS results in moisture absorption from the environment which causes decomposition of the perovskite absorber layer [63,64,65,66,67,68,69,70,71,72,73,74]. Some p-type inorganic materials, including CuSCN, CuI and NiO, have been proposed as promising alternatives to PEDOT:PSS for enhancing the stability of PSCs [75,76,77]. However, the low conductivity of inorganic materials limits the performance of PSCs. Therefore, optimizing the properties of the PEDOT:PSS HTL is crucial in fabricating PSCs with long-term stability. It has been shown that optimization of the PEDOT:PSS HTL layer, such as the pH value, hydrophilicity, work function, surface morphology and electrical conductivity of PEDOT:PSS can improve PCE and stability of PSCs [78,79,80,81,82,83,84,85,86,87,88,89,90,91,92,93,94,95,96,97,98,99,100,101,102,103,104,105,106,107,108].

In this review, we focused on the properties of PEDOT:PSS that affect the stability and performance of PCSs. We summarize the approaches to improve PEDOT:PSS properties and stabilize the long-term performance of PSCs, as well as discuss the corresponding mechanisms of the stability enhancements, and provided potential solutions for further improvement of PSC stability.

## 2. Methods to Improve the PSCs Stability by Tailoring PEDOT:PSS HTL

Many efforts have been made to the modification of the PEDOT:PSS layer for improving the long-term stability of PSCs [78,79,80,81,82,83,84,85,86,87,88,89,90,91,92,93,94,95,96,97,98,99,100,101,102]. Table 1 lists the PCE and long-term stability of PSCs adopting PEDOT:PSS as HTL in previous research work. Generally, modification methods can be classified into three types: doping [61,78,79,80,81,82,83,84,85,86,87,88,89,90,91,92,93,94], post-treatment [62,95,96,97] and using bilayer [98,99,100,101,102]. Furthermore, there are some other methods reported to modify the properties of PEDOT:PSS for improving the device stability, such as, using other dopants to replace PSS [56,103], and developing new processing methods of PEDOT:PSS film [104,105], which are discussed in Section 3.

### 2.1. Doping

PEDOT:PSS is a polyelectrolyte with the positively charged conjugated PEDOT and negatively charged nonconjugated PSS. They are bonded together by the strong Coulombic attractions. PEDOT is conductive and hydrophobic, while PSS is nonconductive, hydrophilic and acidic. Therefore, doping PEDOT:PSS with some compounds can weaken the attraction between PEDOT and PSS, and change the PEDOT chains conformational from coiled to linear, resulting in properties changing. It has been proved that the conductivity of PEDOT:PSS can be increased from 10^−2^ to 10^3^ S/cm by doping an organic compound, such as ethylene glycol (EG), dimethyl sulfoxide (DMSO), an ionic liquid, an anionic surfactant, or dimethyl sulfate, into PEDOT:PSS aqueous solution [23,24,25,26,27,28,29]. Besides conductivity, other properties, such as work function, acidity and hydrophilic properties, can also be modified using the doping method [61,78,79,80,81,82,83,84,85,86,87,88,89,90,91,92,93,94].

**Figure 3:**
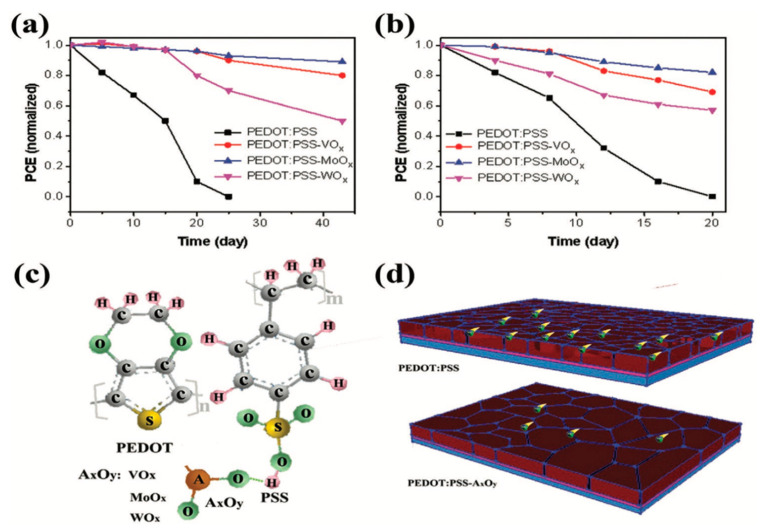
Normalized PCE changes of the devices based on different HTLs in (**a**) glove box and (**b**) air, respectively. Schematic diagram of the effect of the stability of devices based on (**c**) the interaction between metal oxides and PEDOT:PSS and (**d**) the water resistance of perovskite films. Reprinted with permission from ref. [82]. Copyright 2020 John Wiley and Sons.

Due to the sulfonic acid group in PSS, the acidity properties in PEDOT:PSS causes the corrosion of the ITO electrode and deteriorates the stability of PSCs. To solve this problem, some strong bases such as KOH, NaOH and guanidine, have been employed to neutralize acidic PEDOT:PSS at the beginning [106,107,108]. However, the neutralization process by strong base adversely affects the charge-transporting properties of PEDOT:PSS, resulting in reduced device efficiency. Thus, mild bases were adopted later. Wang et al. selected imidazole as an additive to tune the pH value of PEDOT:PSS and alleviate the influence of neutralization processes [78]. The PSCs with this modified PEDOT:PSS HTL show an enhanced PCE of 15.7% with improved long-term stability, which maintains 75% of the original PCE after 14-day storage in ambient condition with a controlled 20% relative humidity. In recent years, Xu et al. doped CuSCN NH_3_ [aq] in PEDOT:PSS to reduce the acidity for alleviating the degradation of MAPbI_3_. The stability of PEDOT:PSS-CuSCN-based PSCs is almost doubled at the same storage condition [79]. Wang et al. doped PEDOT:PSS with either ammonia or ammonium, and the pH value of PEDOT:PSS was turned from 1.9 to 11.0 and 5.0, respectively [80]. The devices with these less-corrosive HTLs exhibit significantly improved stability, resulting in only a 10% decrease of the original PCE after 30-day storage in N_2_ atmosphere. Hytham et al. reported a strategy to tune the acidic nature of PEDOT:PSS by adding urea, where the PCE was increased from 14.4% to 18.8% and the device can maintain 97% of its original PCE after 10-day in ambient air [81]. Duan et al. demonstrated that doping different metal ammonium salts into PEDOT:PSS HTL can significantly enhance the PSCs stability [82]. This is mainly because the oxygen atom in the metal oxide forms a hydrogen bond with the hydrogen ion of PEDOT:PSS, thus inhibiting the corrosion of ITO electrode by the acidity of PEDOT:PSS (Figure 3).

**Figure 4:**
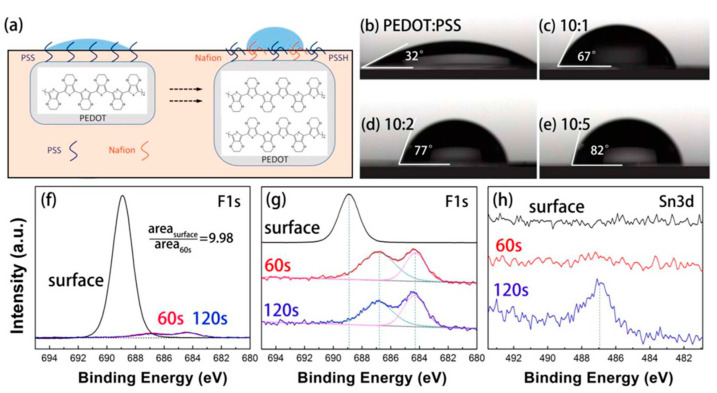
(**a**) Schematic of the structural modification in PEDOT:PSS-Nafion film. (**b**–**e**) Contact angles of water on PEDOT:PSS films with different PEDOT:PSS/Nafion concentration ratios (10:1, 10:2, 10:5). (**f**–**g**) F1s and (**h**) Sn3d X-ray photoelectron spectroscopy (XPS) depth profiling of PEDOT:PSS-Nafion (10:1) film with etching time of 60 s and 120 s. Reprinted with permission from ref. [86]. Copyright 2018 American Chemical Society.

One of the major influence factors of PSCs degradation is the moisture. Moisture in the atmosphere can cause the degradation of perovskite, which is highly sensitive to water. However, PEDOT:PSS is hygroscopic because of the PSS group. Therefore, changing the hygroscopicity of PEDOT:PSS is one of the strategies to improve the PSCs stability. This idea has been demonstrated by doping PEDOT:PSS with hydrophobic material. Huang et al. reported that doping PEDOT:PSS with dopamine (DA) during the polymerization can form less hydrophilic DA-PEDOT. It would alleviate the problem of decomposition reaction of perovskite layer with water. The PCE still retains 85.4% after 28 days in air [83]. Liu et al. used p-type dopant 2,3,5,6-tetrafluoro-7,7,8,8-tetracyanoquinodimethane (F4-TCNQ) and found that the stability of doped solar cells outperformed the reference device, which may be attributed to better interfacial contact and the hydrophobic nature of F4-TCNQ [84]. Another effective way to change the hygroscopic nature of PEDOT:PSS is removing hygroscopic PSS chains in PEDOT:PSS films. Huang et al. doped PEDOT:PSS with a polar organic solvent, dimethyl sulfoxide (DMSO), which can induce segregation of PSSH chains from PEDOT:PSS. Thus, the DMSO doped PEDOT:PSS exhibited lowered hydrophilicity and higher stability as well as enhanced performance [85]. Ma et al. doped Nafion, a hydrophobic perfluorosulfonic-copolymer, into PEDOT:PSS [86]. As shown in Figure 4, the PEDOT:PSS-Nafion film is converted from hydrophilic to hydrophobic by Nafion doping. Moreover, most Nafion chain accumulation is found at the surface of the HTL film, and PEDOT:PSS on the top layer is partially replaced by the newly formed PEDOT:Nafion phase. The Nafion-modified device exhibited enhanced stability which remains 86.6% PCE of the original value after 500 h in air. Such enhanced stability is explained as the enhanced hydrophobicity, mechanical and chemical stability of Nafion polymer concentrated on the surface of PEDOT:PSS film. Recently, Redondo-Obispo reported that adding graphene flakes dispersed into PEDOT:PSS can also improve the device stability [87]. The hydrophobicity of graphene probably blocks undesirable reactions and slows device degradation.

The energy level mismatch between the work functions of PEDOT:PSS (about −5.0 eV) and perovskite active layer (e.g., −5.4 eV for CH_3_NH_3_PbI_3_) results in lower device efficiency and stability. It is because the energy barrier at the interface of PEDOT:PSS/ perovskite not only reduces hole transporting from the perovskite to PEDOT:PSS, but also increases the interface recombination, resulting in low Voc and poor stability [88,89]. Thus, modification of the electronic properties of PEDOT:PSS is important for improving the device stability. To achieve better energy level match, Zuo and Ding doped polymer electrolyte PSS-Na into PEDOT:PSS to tune the work function and obtained an outstanding V_OC_ of 1.52 V in the CH_3_NH_3_PbI_3_-based PSCs [88]. The PSCs with modified PEDOT:PSS HTL can maintain more than 85% of its original PCE after 60-day storage in N_2_ atmosphere. Later, Tang et al. doped PEDOT:PSS with a perfluorinated ionomer (PFI) to reduce the mismatch of energy levels between perovskite active layer and PEDOT:PSS HTLs [90]. The performance of devices with modified PEDOT:PSS HTLs does not degrade even after 300 s operation at maximum power point, whereas the devices with PEDOT:PSS immediately decay seriously under the same testing conditions.

**Figure 5:**
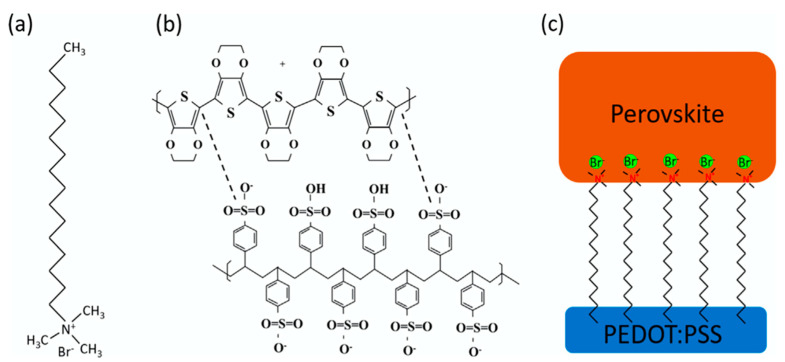
Molecule structures of (**a**) CTAB and (**b**) PEDOT:PSS. (**c**) The connections of CTAB to PEDOT:PSS and perovskite layer at their interface. Reprinted with permission from Ref. [91]. Copyright 2019 Elsevier.

Another effective way to alter the electronic properties of PEDOT:PSS is to mix surfactant into PEDOT:PSS. Shin et al. added Triton X-100 (TX), a nonionic surfactant, into PEDOT:PSS HTL [90]. The electronic structure of PEDOT:PSS was changed significantly with the TX concentration. The concentrated TX on the surface reduces the semi-metallic property of PEDOT:PSS, resulting in improved PSCs performance and stability. Zhu et al. doped PEDOT:PSS with a common surfactant Cetyl trimethyl ammonium bromide (CTAB) [91]. As shown in Figure 5, Br^−^ in CTAB would diffuse into the perovskite layer, leading to higher Voc and the ammonium group in CTAB participated in the passivation process. As a result, the device using CTAB-doped PEDOT:PSS exhibited enhanced PCE and still retained over 75% of the initial PCE after being exposed at the ambient condition (20–40% humidity, 10–30 °C) for 30 days. The improvement of long-term device stability was attributed to the larger grain size of perovskite and passivation process.

**Figure 6:**
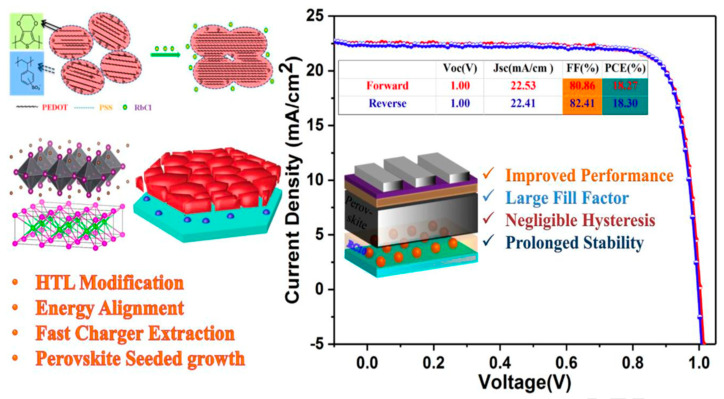
(**Left**) Schematic of structural modification and the process of hole extraction and transport in PEDOT:PSS with the addition of RbCl. (**Right**) J-V curves of the mixed perovskite using RbCl- doped PEDOT:PSS. Reprinted with permission from ref. [61]. Copyright 2018 Elsevier.

The low conductivity of PEDOT:PSS induces interface recombination and therefore causes a detrimental effect on the device stability. The electrical conductivity of the PEDOT:PSS can be increased through the doping method, which can also optimize the morphology of PEDOT:PSS and enlarge the grain size in the perovskite layer. Large grain size indicates better crystallinity and fewer grain boundaries, where the humidity could penetrate the perovskite film. The fewer grain boundaries of the perovskite layer contribute to higher humidity stability [92]. Li et al. demonstrated that doping PEDOT:PSS with sodium benzenesulfonate (SBS) can improve the device performance of inverted PSCs with enhanced stability. It was observed that the modified PEDOT:PSS was more smooth and had higher conductivity, leading to better crystallinity in the perovskite film. The PCE can retain 90% after 20 days storage in air [92]. Zhou et al. found that ionic liquid can also increase the conductivity of PEDOT:PSS. The PSCs with ionic liquid doped PEDOT:PSS film can retain 85% of initial PCE after 35-day storage in air with 60% humidity without encapsulation [93]. Recently, Alishah et al. used Zn as an additive in PEDOT:PSS solution to successfully improve the hole mobility and electrical conductivity of PEDOT:PSS film and reduce the perovskite trap density, leading to improved environmental stability with 91% of initial PCE for 168 h [94]. Another effective strategy is to dope PEDOT:PSS with inorganic halometallate to suppress the charge recombination and increase the stability. Liu et al. doped dispersed rubidium chloride (RbCl) in the aqueous PEDOT:PSS solution [61] (Figure 6). With systematic characterizations, they found that the RbCl could cause phase segregation of PEDOT:PSS and increase its nanocrystal size, result in enhanced hole transport capability, electrical conductivity and work function. Moreover, RbCl has similar polyhedral structure and lattice parameters, which can be functioned as growing sites to guide the seed-mediated growth of the perovskite, leading to better crystallinity and a more compact and smooth perovskite layer. Consequently, the trap densities can be remarkably reduced, resulting in improved device stability. The PSCs based on the RbCl doped PEDOT:PSS HTL retain 78.17% of initial PCE for 120 h.

Doping method can weaken the Coulombic attractions between PEDOT and PSS to improve the properties of PEDOT:PSS HTL, such as conductivity, work function, acidity and hydrophilic properties. However, excessive dopant would destroy the morphology of PEDOT:PSS and hinder the hole transport and extraction [79,81,84,86,88,90,91,92,93]. Thus, it is important to develop appropriate dopants and control the doping ratio when using a doping method to modify the drawbacks of PEDOT:PSS.

### 2.2. Post-Treatment

Another common method to improve the PSCs stability is post-treatment on PEDOT:PSS HTL film. Like the doping method, post-treatment can also weaken the attraction between PEDOT and PSS, resulting in the phase segregation of PSSH from PEDOT:PSS and conformational change of the PEDOT chains [30,31,32,33,34,35,36]. Similar conductivity enhancement was also observed by treating PEDOT:PSS films with polar organic compounds, acids, salts, zwitterions, or cosolvents [30,31,32,33,34,35,36]. The difference is that post-treatment mainly affects the surface of the PEDOT:PSS layer. It has been reported that the hygroscopic PSS at the PEDOT:PSS surface can be partially removed by post-treatment, leading to a reduction of acidity and water absorption [30,31,32,33,34,35,36,62,95,96,97]. In addition, a non-wetting surface and more compact structure of PEDOT:PSS is suitable for the growth of larger perovskite crystalline which further enhances the device performance and stability [62,95,96,97].

**Figure 7:**
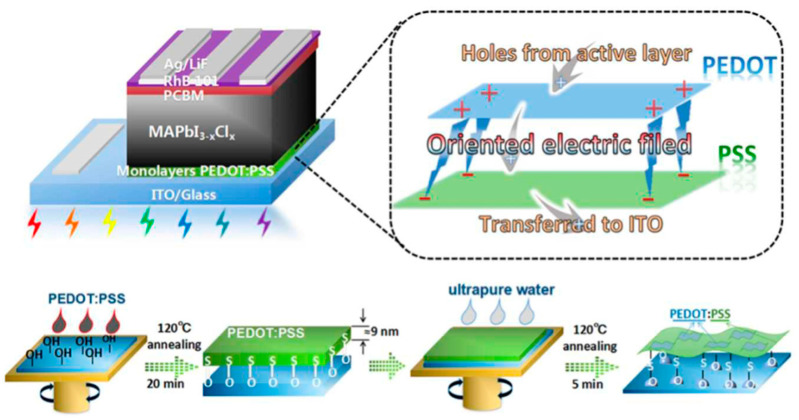
Tentative model explaining the high working efficiency of monolayer PEDOT:PSS film and major fabricating steps. Reprinted with permission from ref. [62]. Copyright 2018 The Royal Society of Chemistry.

Most post-treatment research mainly focused on the solvent treatment of the PEDOT:PSS layer. For instance, Luo et al. reported that spin-coating a graphene-oxide (GO) solution on top of the PEDOT:PSS layer can remove the unnecessary PSS component, improve the wettability of PEDOT:PSS layer and inhibit the carrier recombination at the interface between the PEDOT:PSS and perovskite layers. Therefore, the PSCs stability was significantly improved with the PCE remaining at 83.5% of the initial values after aging for 39 days in air [95]. Kanwat et al. studied the function of ethylene glycole (EG) on conductivity improvement of WOx doped PEDOT:PSS films. PEDOT:PSS films with multiple EG treatments exhibit high conductivity and improved thermal stability. Thus, the PCEs with modified PEDOT:PSS HTL showed significant thermal and environmental stability [96]. Later on, Reza et al. found that two-step sequential solvent treatment of PEDTO:PSS film with EG and methanol remarkably increased the hydrophobicity and conductivity of the film, resulting in the formation of highly crystalline smooth perovskite films with larger grains. The formation of highly conductive HTL and efficient charge extraction with high-quality perovskite films led to a significant improvement of device stability and efficiency. The devices using treated PEDOT:PSS could maintain up to 65% of the initial efficiency after 350 h storage in air [97]. Kuan Sun group [62] reported a simple post-treatment to achieve PEDOT:PSS monolayers by water rinsing the spin-coated PEDOT:PSS films (Figure 7). The PSCs using water rinsed PEDOT:PSS as HTL showed an increase in PCE from 13.4% to 18.0% and an improved stability in air. The better device performance is attributed to the internal electric field originated from the PEDOT/PSS bilayered structure that facilitates hole extraction. Moreover, the oriented arrangement of PEDOT:PSS monolayers endow stronger hydrophobicity and higher work function, resulting in the improvement of stability and PCE in an ambient environment.

Post-treatment is a convenient method that successfully removed surface PSS to reduce the acidic and hydrophilic nature of PEDOT:PSS and thus increased the device stability. However, the materials used for post-treatment remained on the surface of PEDOT:PSS may cause decreases in device performance [97]. Thus, the treating materials which can be easily removed and mild procedures to remove excess treating materials should be further developed.

### 2.3. Bilayer

Modification of the interface between PEDOT:PSS and perovskite layers is one of the approaches for increasing device efficiency and stability. Besides modifying the surface of PEDOT:PSS film by post-treatment, many trials have been conducted on the use of bilayer HTL to modify the interface and provide a synergetic effect on the hole transporting ability [98,99,100,101,102]. Therefore, other hybrid materials such as metal oxides [98,99], graphene oxide [101], and small molecules [100,102] can be used as an interlayer between PEDOT:PSS and perovskite to reduce the influence of the acidic and hydrophilic PEDOT:PSS on the quality of perovskite.

**Figure 8:**
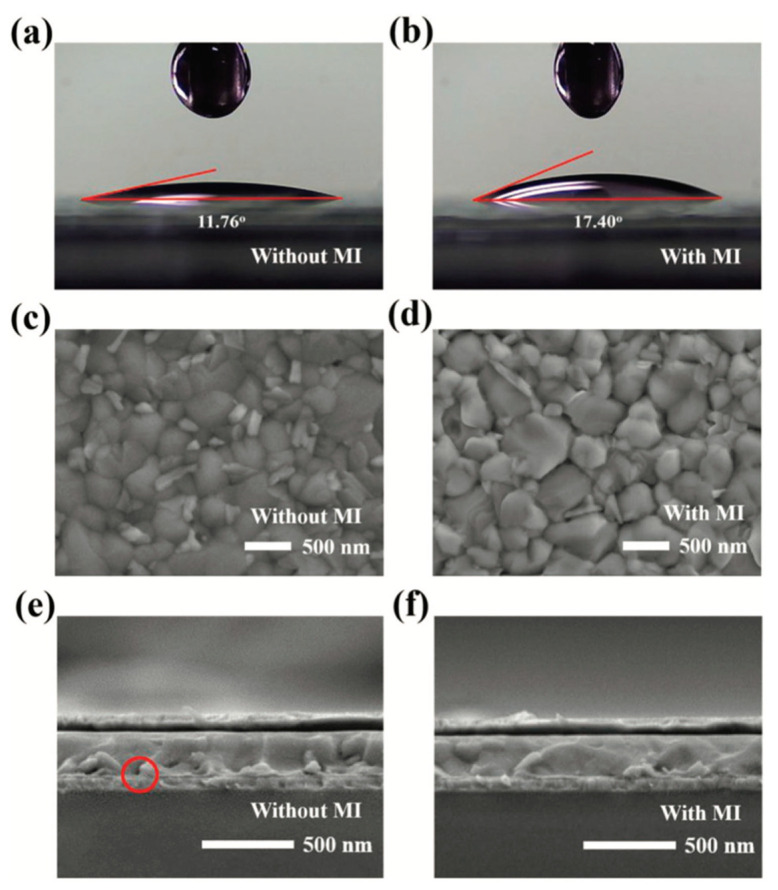
Contact angles of the PEDOT:PSS films (**a**) without and (**b**) with 2-Mercaptoimidazole modification layer. Top-view SEM images of perovskite films deposited on ITO substrates coated with (**c**) unmodified PEDOT:PSS and (**d**) the 2-Mercaptoimidazole-modified PEDOT:PSS (PEDOT:PSS MI). Cross-sectional SEM images of PSCs (**e**) without and (**f**) with 2-Mercaptoimidazole modification layer. Reprinted with permission from ref. [102]. Copyright 2020 John Wiley and Sons.

Wang et al. incorporated vanadium pentoxide (V_2_O_5_) and PEDOT:PSS bilayer (PVO) for better charge transport in the device [98]. The devices with bilayer are more stable than PEDOT only ones, retaining 70% of their initial PCE even after 35-day testing. The reason for stability enhancement is because the V_2_O_5_ layer prevents the ITO from direct contact with the PEDOT layer thereby reducing the corrosive degradation of the ITO and hence contributing to the improved stability of the PVO device. Subsequently, Xu et al. added a solution processed VOx interlayer to modify PEDOT:PSS for inverted PSCs, which could modify the Fermi level, acidic and hygroscopic properties of pristine PEDOT:PSS HTL, leading to improved device performance and stability [99]. Recently, Ma et al. incorporated NPB, a small-molecule, at the PEDOT:PSS/perovskite interface. This buffer layer can alleviate the reduction reaction between perovskite precursor and PEDOT:PSS, achieve well-matched energy level alignment and improve perovskite film quality. Most importantly, because of the excellent UV-light and moisture resistance of NPB, the modified device shows superb long-term stability under ambient atmosphere and UV-light soaking [100]. Mann et al. fabricated bilayered PSCs containing PEDOT:PSS and sulfonic acid functionalized graphene oxide (SrGO) as the HTL. The device using PEDOT:PSS/SrGO exhibits excellent long-term stability in ambient air condition probably due to the hydrophobicity and chemical stability of SrGO [101]. Wang et al. reported that the stability and performance of PSCs can be enhanced by employing an alcohol-soluble small molecule, 2-mercaptoimidazole (MI), at the interface between PEDOT:PSS and perovskite layers [102]. As shown in Figure 8, the MI modified PEDOT:PSS films are more hydrophobic and assists better perovskite film formation. The reduced interface defects and improved crystallization quality of perovskite films are the major reasons for the improvement of device stability.

Bilayer structure of HTL is an efficient method to modify the interface of PEDOT:PSS/ perovskite. However, flexible and solution processable buffer layers with appropriate HOMO levels still need to be further explored.

## 3. Other Methods to Improve the PSCs Stability by Tailoring PEDOT:PSS Layer

**Figure 9:**
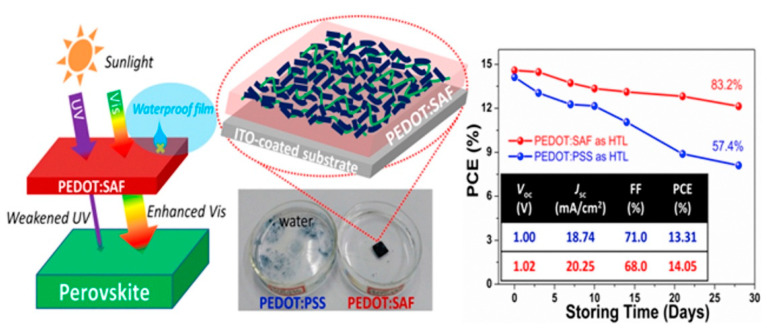
Schematic diagram showing the reduced UV radiation on pervoskite layer due to absorbtion by the PEDOT:SAF-based HTL. Schematic of proposed structures inside PEDOT:SAF film on ITO-coated substrate. Stability of the PSCs using PEDOT:SAF and PEDOT:PSS as the HTLs. Reprinted with permission from ref. [56]. Copyright 2017 Elsevier.

Many other ameliorative techniques have been developed to improve the device stability using various approaches. Since the aforementioned drawbacks of PEDOT:PSS are almost all related to PSS, some groups focus on exploring other dopants to replace PSS. Jiang et al. reported a facile solid-state synthesis of conducting polymer PEDOT through an in-situ solid-state polymerization from an inexpensive monomer 2,5-dibromo-3,4-ethylenedioxythiophene (DBEDOT) [103]. The devices using DBEDOT as HTL remained over 80% of their initial PCEs after 720 h storage time. Yu et al. reported a new PEDOT-based HTL adopting sulfonated acetone-formaldehyde (SAF) as the dopant to enhance the PSCs stability (Figure 9) [56]. PEDOT:SAF exhibited extremely reduced acidity with pH value at around 6 and significantly improved conductivity of 3.12 S/cm. The PEDOT:SAF-based PSC was highly stable with 83.2% of its initial PCE after 28 days of storage. The outstanding characteristics of PEDOT:SAF especially the excellent waterproofness and UV-absorptivity were considered as the key factors for the enhanced device stability.

Some groups focus on new fabricating methods of PEDOT:PSS film to improve its physical and electrical properties. Erazo et al. deposited PEDOT:PSS layers by an alternative electrochemical (EC) route that offers precise synthesis control, scale-up potential and enhanced cell stability [104]. The EC-PEDOT:PSS significantly improved the stability of the cells, allowing the devices to maintain 90% of their average efficiency after 15 days. The improved stability is probably related to the lower acidic PSS content in the EC-PEDOT:PSS films. The electrodeposited films present a more hydrophobic nature. Chen et al. proposed a facile strategy of cryo-controlled quasi-congealing spin-coating to improve the PEDOT:PSS quality to a new extent [105]. This smooth and passivated PEDOT:PSS film facilitates the growth of highly crystalline perovskite and the achievement of better interfacial contact between them, which not only enhance the device efficiency, but also greatly improve the mechanical stability of PSCs under repeated deformation.

Recently, some studies have been reported on incorporating two-dimensional (2D) materials into PSCs to improve the device performance and stability [40,110,111,112]. 2D materials, such as graphene and its derivatives, black phosphorus, and transition-metal dichalcogenides, with unique van der Waals structure and properties, can increase perovskite film quality and reduce defect states, resulting in better PCE and stronger stability. Therefore, 2D materials have the potential to be incorporated into PEDOT:PSS/PSCs, which could be an interesting research direction for researchers to explore in the future.

## 4. SWOT Analysis

Here, we provide a brief Strengths-Weaknesses-Opportunities-Threats (SWOT) analysis of PEDOT:PSS used in PSCs compared with other HTL materials.

Strengths: (1) PEDOT:PSS is a low cost material; (2) PEDOT:PSS is commercially available; (3) PEDOT:PSS can be dispersed in water and some organic solvents and high-quality PEDOT:PSS films can be readily coated on substrates through conventional solution processing techniques; (4) Methods for the deposition/fabrication of PEDOT:PSS are energy efficient. (5) The electrical properties of PEDOT:PSS can be tailored for specific applications; (6) PEDOT:PSS is flexible and lightweight; (7) PEDOT:PSS film is transparent in the visible range; (8) PEDOT:PSS is nontoxic.

Weaknesses: (1) The acidic nature of PEDOT:PSS will corrode the electrode materials, leading to poor long-term device stability; (2) the hygroscopic nature of PEDOT:PSS can cause decomposition of the perovskite absorber layer. (3) The conductivity of untreated PEDOT:PSS film is low and need additional treatments.

Opportunities: (1) There is still some room for improving the conductivity of PEDOT:PSS to replace the high cost and rigid ITO electrode; (2) The peculiarities of PEDOT:PSS make them the most suitable choice for flexible electronics and wearable devices.

Threats: Other emerging HTL materials may overcome the main limitations of PEDOT:PSS.

## 5. Conclusions

In conclusion, we summarized the recent efficient strategies for improving the long-term stability of PEDOT:PSS-based PSCs. The characteristics of PEDOT:PSS can be tailored to improve the stability of PSCs by doping, post-treatment and bilayer formation. To neutralize the acidity of PEDOT:PSS solution and to reduce the hydrophilicity of PEDOT:PSS are the main strategies to tackle the degradation problem of PSCs. It is important to select appropriate materials and modify methods for further PSCs stability development.

## Figures and Tables

**Table 1:** The long-term stability of PSCs with PEDOT:PSS as HTL.

Method	Materials	Perovskite Materials	PCE (%)	Stability	Ref.
Doping	Imidazole	MAPbI_3_	15.7%	75% for 14 days, 20% humidity	[78]
	CuSCN /NH_3_ (aq)	MAPbI_3_	15.3%	71% for 175 h	[79]
	Ammonia	MAPbI_3-x_Cl_x_	13.38%	90% for 30 days in N_2_	[80]
	Urea	MAPbI_3_	18.8%	97% for 10 days, 35% humidity	[81]
	metal oxides	MAPbI_3_	19.64%	90% for 45 days in N_2_, 80% for 20 days in air	[82]
	Dopamine	MAPbI_3_	16.4%	85.4% for 28 days	[83]
	F4-TCNQ	MAPbI_3-x_Cl_x_	17.22%	75% for 150 h, 40% humidity	[84]
	DMSO	MAPbI_3_	16.7%	83% for 590 h	[85]
	Nafion	MAPbI_3_	16.72%	86.6% for 500 h, 30–50% humidity	[86]
	graphene flakes	MAPbI_3_	4%	Stable for one weak	[87]
	PSSNa	MAPbI_3_	15.56%	>85% for 60 days in N_2_,	[88]
	PFI	FA_0.6_MA_0.4_Sn_0.6_Pb_0.4_I_3_	15.85%	Stable for 300 s	[89]
	Triton X-100	MAPbI_3_	16.23%	80% for 500 h	[90]
	CTAB	MAPbI_3_	12.53%	75% for 30 days, 20–40% humidity	[91]
	SBS	MA_0.8_FA_0.2_PbI_3-x_Cl_x_	19.41%	90% for 20 days	[92]
	EMIC ionic liquid	MAPbI_3_	20.06%	85% for 35 days, 60% humidity, 87% after 80 °C for 24 h	[93]
	Zn	MAPbI_3_	13.2%	91% for 168 h	[94]
	RbCl	MA_0.7_FA_0.3_Pb(I_0.9_Br_0.1_)_3_	18.3%	78.17% for 120 h, 50% humidity	[61]
Post-Treatment	GO	MAPbI_3_	15.34%	83.5% for 39 days, 15% humidity	[95]
	WOx doped, EG treated	MAPbI_3_Cl_3-x_	12.69%	thermal stable at 250 °C	[96]
	EG and MeOH	MAPbI_3_	18.18%	65% for 350 h, 45% humidity	[97]
	Water	MAPbI_3-x_Cl_x_	18.0%	50% for 240 h in air	[62]
Bilayer	V_2_O_5_	MAPbI_3_	15%	95% for 18 days	[98]
	VO_x_	MAPbI_3_	14.22%	77% for 15 days, 40% humidity	[99]
	NPB	MAPbI3	18.4%	70% for 20 days, 30±5% humidity	[100]
	SrGO	MAPbI3	16.01%	85% for 30 days	[101]
	MI	FA_0.2_MA_0.8_PbI_3-x_Cl_x_	20.68%	80% for 600 h, 50% humidity	[102]

## Data Availability

Data sharing not applicable.
